# Benchmarking Active Learning Protocols for Ligand-Binding Affinity Prediction

**DOI:** 10.1021/acs.jcim.4c00220

**Published:** 2024-03-06

**Authors:** Rohan Gorantla, Alžbeta Kubincová, Benjamin Suutari, Benjamin P. Cossins, Antonia S. J. S. Mey

**Affiliations:** † School of Informatics, University of Edinburgh, Edinburgh EH8 9AB, U.K.; ‡ EaStCHEM School of Chemistry, University of Edinburgh, Edinburgh EH9 3FJ, U.K.; § Exscientia, Schrödinger Building, Oxford OX4 4GE, U.K.

## Abstract

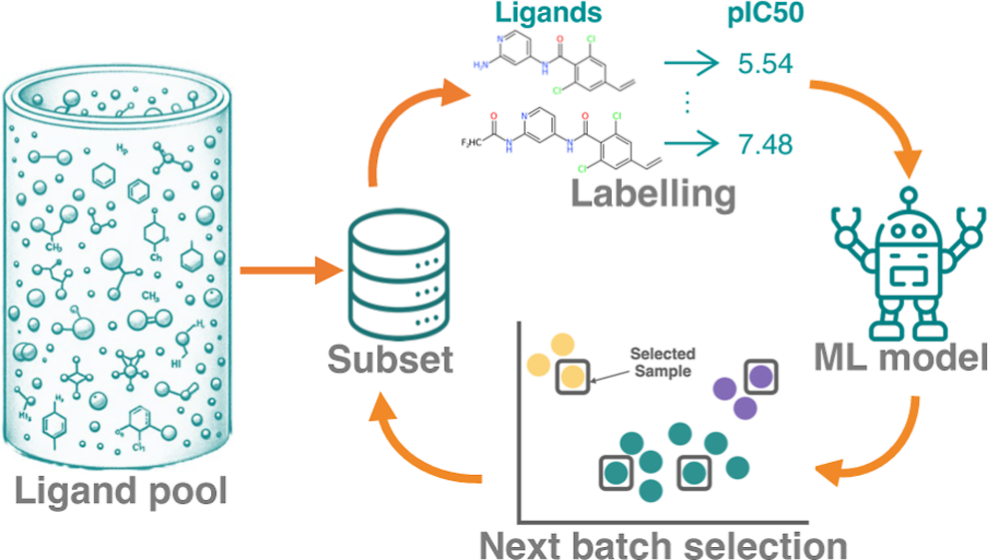

Active learning (AL) has become a powerful tool in computational drug discovery, enabling the identification of top binders from vast molecular libraries. To design a robust AL protocol, it is important to understand the influence of AL parameters, as well as the features of the data sets on the outcomes. We use four affinity data sets for different targets (TYK2, USP7, D2R, Mpro) to systematically evaluate the performance of machine learning models [Gaussian process (GP) model and Chemprop model], sample selection protocols, and the batch size based on metrics describing the overall predictive power of the model (R2, Spearman rank, root-mean-square error) as well as the accurate identification of top 2%/5% binders (Recall, F1 score). Both models have a comparable Recall of top binders on large data sets, but the GP model surpasses the Chemprop model when training data are sparse. A larger initial batch size, especially on diverse data sets, increased the Recall of both models as well as overall correlation metrics. However, for subsequent cycles, smaller batch sizes of 20 or 30 compounds proved to be desirable. Furthermore, adding artificial Gaussian noise to the data up to a certain threshold still allowed the model to identify clusters with top-scoring compounds. However, excessive noise (<1σ) did impact the model’s predictive and exploitative capabilities.

## Introduction

Active learning (AL) is a semisupervised machine learning (ML) method, which makes use of a model to guide the selection of new samples to label unlabeled data of interest in an iterative process. In the context of computational drug discovery, this method has been used to identify potent inhibitors in small-molecule libraries at a fraction of the cost associated with a systematic potency screen.^1−3^

The identification of drug candidates requires a balance of novelty from exploring a new chemical space with optimization of known leads by means of small substitutions. The tension between exploration and exploitation is reflected in AL campaigns,^2^ and combinations of the two strategies are common, although the exact procedures vary widely.^4,5^ An exploration strategy aims to select samples that are representative of the underlying chemical space in order to construct a good potency model.^6^ Exploitative strategy, on the other hand, aims to retrieve a high amount of potent compounds by means of a greedy acquisition based on the predicted binding affinity.

Traditionally, AL has been considered in the late stages of lead optimization to select compounds for synthesis. Retrospective studies on affinity data were able to retrieve top binders by using information from a small subset of the data.^4,5^ The procedure was also combined with an automated synthesis setup to select products from a matrix resulting from two types of reagents.^7^ Despite being successful, the throughput in these approaches was low (tens of compounds selected for labeling), and the small sizes of the libraries employed (hundreds of compounds) are often associated with a restricted chemical space.

Computational potency prediction methods have evolved over the last four decades from traditional docking,^8,9^ alchemical free energy (AFE) techniques^10,11^ to more recently ML approaches.^12−14^ However, the use of AL applications together with computational potency estimation such as virtual screening^15−18^ or relative binding free energy (RBFE) calculations using molecular dynamics simulations^19−23^ only emerged in the past 8 years, driven by the increase in automation and throughput of computational tools for drug discovery. In these cases, 100s to 1000 compounds are selected out of pools containing up to 100,000 samples. The sheer size of the compound pool goes hand in hand with a high degree of diversity compared to low-throughput use cases, putting more strain on the AL pipeline and necessitating a careful selection of molecular features, ML models, and acquisition methods.^15,19,22,23^ In addition to the challenge posed by data set sizes and diversity, using RBFEs or docking scores in lieu of experimental binding affinities introduces errors of systematic and stochastic nature, which are often not well characterized in advance.

Although AL presents an opportunity to quickly identify an active chemical space in large ligand pools, a routine application of this method in the pharmaceutical industry requires establishing a robust protocol that is transferable between different data sets. Previous AL studies used RBFE as their labeling tool of choice and only investigated ligands for a single target.^19,21−23^ The scarcity of large public RBFE data sets and the cost and difficulty of generating them are additional hurdles for establishing robust AL–RBFE benchmarks. Furthermore, none of the studies mentioned above considered cost as a factor in the selection of a protocol, resulting in very large initial batches or exploration phases.^19,22^ They also compared protocols that require a variable amount of RBFE data.^21^ The difference between data sets, their sizes and generation procedures, as well as applied AL protocols, and different combinations of metrics to evaluate the performance of AL make it difficult to compare literature protocols and identify best-practice approaches.

The aim of this study is to evaluate AL protocols in a rigorous manner by using four publicly available data sets for benchmarking. The main focus is on the AL design and not on the method used for labeling data. Different strategies for labeling are possible, such as docking,^9^ AFE methods (relative or absolute methods),^10^ experimental measurements, or an ML property prediction model.^14^ How to choose best labeling strategies and how to mix different ones will not be evaluated here. Instead, we use four different data sets, where we already have labels provided. The chosen data sets differ in their protein targets, kind of potency measurement (Δ*G* from RBFE or experimental *K*_i_/IC_50_), size (600 to 10000 samples), and degree of diversity. The Tyk2 data set has labels from predicted RBFE values (Δ*G*), which are converted to binding affinities (*K*_i_), while all other data sets comprise experimental values. Our primary objective is to investigate how the diversity and size of data sets affect the efficacy of AL. We use a wide range of metrics to gain a holistic perspective, ranging from conventional regression metrics, such as R2, to assess the overall performance of the ML model, to Recall and F1 scores for the top 2%/5% binders to assess the exploitative capabilities of a model and the degree of exhaustion of the active chemical space. To investigate the benefit of pretrained model architectures for AL, we compare a fine-tuned Chemprop (CP) model with Gaussian process (GP) regression, which is a common choice for AL.^21,22^ Finally, the total number of acquired samples is always kept constant throughout all experiments to compare AL protocols at a fixed cost. With our prelabeled data, we do not have a choice how to label the data, but any labeling method can be used, such as a docking score, an experimental value, or an RBFE calculation. However, mixing different methods will require care in accounting for accuracy or trustworthiness of the labeling method used and is beyond the scope of this work.

The paper is structured as follows. In the Methods section, we give a detailed overview of the data sets and provide details of the AL procedure, ML models, and metrics. In the Results and Discussion section, the GP and CP models are first benchmarked to assess differences in their predictive power between data sets. Next, we compare AL procedures by varying the size of the initial batch and the method for its acquisition. Thereafter, we identify an optimal batch size for cycles that come after the initial batch(es). Finally, we assess the robustness of AL toward stochastic noise in the potency data.

## Methods

### Data Sets

**Figure 1:**
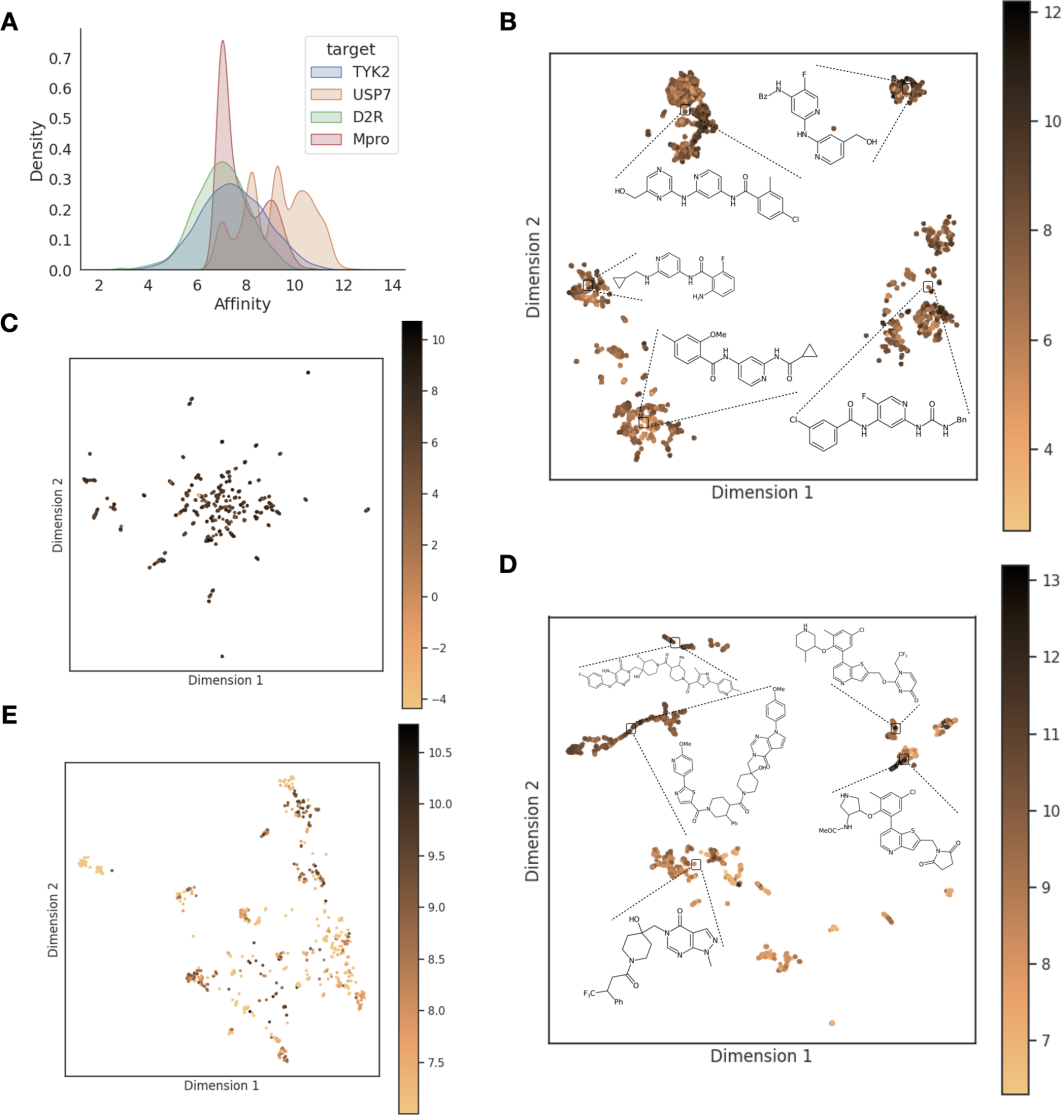
Distribution of affinity scores and UMAP projections for four protein targets. (A) Kernel density estimation plot illustrating the distribution of affinity scores for each target data set: p*K*_i_ values for TYK2 and D2R and pIC_50_ values for USP7 and Mpro. The standard deviations (1 σ) for the pKi/pIC_50_ values are as follows: 1.36 for TYK2, 1.31 for USP7, 1.44 for D2R, and 0.91 for Mpro. (B) UMAP projection of the TYK2 data set with overlaid cluster centroid compounds. (C) UMAP visualization of the D2R data set. (D) UMAP projection of the USP7 data set with overlaid cluster centroid compounds. (E) UMAP representation of the Mpro data set.

We used four publicly available binding affinity data sets that encompass the following protein targets: Tyrosine Kinase 2 (TYK2); a G protein-coupled receptor target, Dopamine Receptor D2 (D2R); and two proteases, Ubiquitin-Specific Protease 7 (USP7) and SARS-CoV-2 Main Protease (Mpro). Table 1 provides the number of ligands in each data set as well as other information relevant for subsequent AL experiments. Figure 1A shows the distribution of the measured affinities associated with each data set. All the data sets used in our study are accessible at https://github.com/meyresearch/ActiveLearning_BindingAffinity.

**Table 1:** Summary of Data Set Characteristics for Protein Targets Used in Our Study^a^

target	ligands	binding measure	% data for AL	top 5%	top 2%
TYK2	9997	p*K*_i_	3.6	500	200
USP7	4535	pIC_50_	7.9	227	90
D2R	2502	p*K*_i_	14.4	125	50
Mpro	665	pIC_50_	54.1	33	13

a Total number of ligands, the binding measure used for training and inference, the percentage of data utilized for AL based on a consistent sample of 360 compounds acquired over AL cycles, and the count of compounds in the top 5% and top 2% fraction of the dataset.

The TYK2 data set was derived from the work of Thompson et al.,^21^ which focused on optimizing the AL methodologies for RBFE calculations. This data set comprises 10000 congeneric molecules targeting the TYK2 kinase, all of which were synthesized using an aminopyrimidine core scaffold. The data set was initially populated with 573 TYK2 inhibitors, which were subsequently decomposed into unique R-groups at three attachment points. These groups were then combinatorially assembled to create an initial library of 203406 unique compounds, which was then filtered down to 10000 molecules based on a set of “drug-like” properties. ΔΔ*G* values obtained from RBFE calculations were converted to p*K*_i_ values, providing a more interpretable metric for binding affinity, using
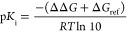
1where p*K*_i_ is the negative logarithm of the inhibition constant, which is a measure of the binding affinity of a ligand for its target (TYK2). ΔΔ*G* is the free energy difference of binding between a reference compound and a second compound. Δ*G*_ref_ is the absolute binding free energy of the reference compound (−47.778 kJ/mol for TYK2), *R* is the universal gas constant, approximately equal to 8.314 J/(mol·K), and *T* is the absolute temperature. In Figure 1B, the Uniform Manifold Approximation and Projection (UMAP)^24^ of the TYK2 data set shows clear clusters that capture variations in R-groups attached to the core scaffold. We can see that most of the active compounds are located within the two upper clusters. Figure S1 in the Supporting Information highlights that the majority of the top 2% binders are situated here.

The USP7 data set was curated by Shen et al.,^25^ with the primary objective of building a classification model to distinguish active from inactive inhibitors. The SMILES of over 4000 ligands together with their experimental affinities including *K*_i_, *K*_d_, and IC_50_ were collected from ChEMBL.^26^ Duplicate SMILES were aggregated into unique entries using the Open Babel package 2.3.1^27^ by Shen et al.^25^ All experimental results with varying units were converted to IC_50_ values for each SMILES, which, for the scope of our study, were translated to pIC_50_ values. As evident from Figure 1A, the USP7 data set exhibits multiple assay minima. Moreover, the UMAP visualization with cluster centroids in Figure 1D highlights the data set’s diverse core scaffolds and R-groups, showing the presence of heterogeneity. However, scaffolds tend to be well-preserved within a cluster. A significant portion of the top active compounds can be found in the upper regions of the UMAPs.

The D2R data set is a subset of the ACNet data set,^28^ which was curated from the ChEMBL^26^ database (version 28) on 190 targets to study the performance of ML models on data from activity cliffs. Zhang et al.^28^ categorized matched molecular pairs as activity cliff if the difference in potency is p*K*_i_ ≥ 2. We selected the D2R target due to the high number of associated activity data, making it particularly suitable for our study. The ACNet data set was constructed by screening over 17 million activities, only retaining compounds tested against single human targets in direct interaction binding assays and filtering data with low assay confidence. The data set uses assay-independent equilibrium constants (p*K*_i_) as the measure of potency. We retained the p*K*_i_ values and averaged over duplicate entries with the same SMILES to ensure data consistency, leading to a reduction from 4121 to 2502 entries. From the UMAP in Figure 1D, the D2R data set appears to encompass a heterogeneous assortment of compounds, with a large number of congeneric series of 10–20 compounds each. The absence of distinct clusters and the dispersed distribution of binding scores suggest intricate structure–activity relationships.

The Mpro data set is part of the COVID Moonshot project,^29^ which focuses on the development of inhibitors for the SARS-CoV-2 main protease. The data set provides experimental pIC_50_ values, which are an amalgamation derived from single enantiomers and racemic mixtures. The project consists of several design cycles (sprints) in which medicinal chemists select compounds for synthesis out of a database with public submissions. The UMAP depicted in Figure 1E shows a diverse composition of the Mpro data set. The absence of distinct clusters and dispersed distribution of top active compounds highlight its intricate nature. Importantly, with only 665 compounds, this data set is significantly smaller than the other data sets in our study, offering a distinct context for AL pipeline investigations in low-data settings.

### AL Protocols

**Figure 2:**
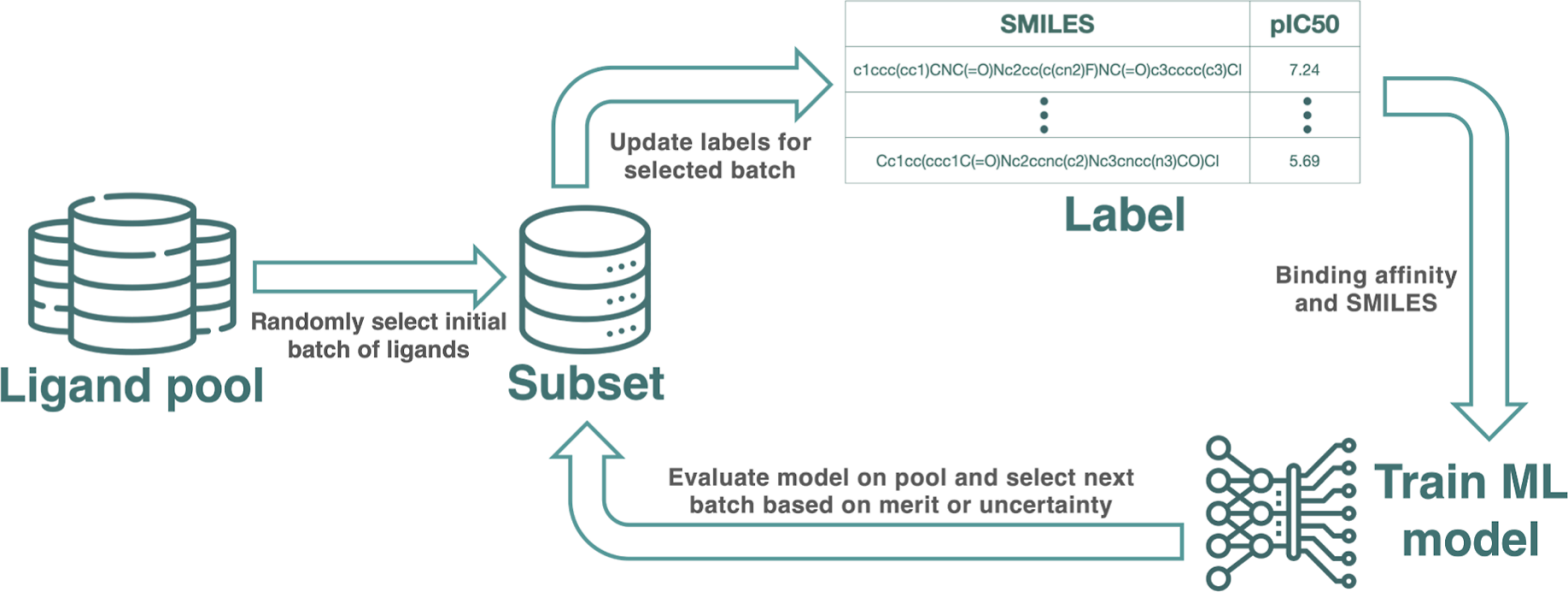
Schematic overview of the AL pipeline. An AL cycle begins with a randomly chosen batch from the available pool of compounds, followed by labeling and model training on the subset with labels. The subsequent batch for the next AL cycle is strategically chosen based on model predictions and uncertainties using exploration, exploitation, or random strategies.

AL is a ML paradigm designed to optimize the selection of samples for training models (Figure 2). It is particularly useful for problems where labels can be calculated for every data point, but the associated computational cost is high, as is the case for RBFE calculations or testing the data point in the lab. The key difference from conventional ML consists of splitting the training process into several AL cycles such that a model trained on a subset of samples informs the selection of the next batch to be added to its training set.

The initial batch of compounds was selected at random, as this is a common strategy in previously published studies.^15,21^ This choice also ensures that the distribution of the training data matches the pool used for inference, which is not the case in diversity-based selection methods.

In a real AL use case, labels for the selected compounds can be acquired by means of RBFE calculations. We take the advantage of experimental potency values from the literature instead of RBFE calculations. This provides robust insight into AL on retrospective data.

A model is then trained on a subset of labeled samples and used to make predictions on the unlabeled data. On the basis of these predictions, a strategic subset of samples is selected for labeling, often employing strategies aimed at either “exploration” of the chemical space or “exploitation” of promising regions within it. These newly labeled samples are then incorporated into the training set for the next AL cycle.

In each AL cycle, we employ one of the strategies for sample selection: random sampling, exploration, and exploitation. Random selection involves choosing compounds arbitrarily from the remaining data. Exploitation focuses on selecting compounds with the highest predicted potency, thereby exhausting high-potency areas in the chemical space. Exploration selects compounds with the highest prediction uncertainty, aiming to sample broadly across the chemical space to gain a more global understanding, thereby potentially identifying new promising areas in the chemical space.

To ensure a fair evaluation for different targets, we always acquire a total of 360 compounds for labeling over the whole AL procedure, irrespective of the data set, model, or selection protocol. The number of AL cycles is always adjusted to fit this total depending on the batch sizes. Additionally, to account for variability, each experiment was conducted three times by using different seeds for the initial batch selection.

### ML Models

#### GP Regression

We chose GP regression^30^ for its ability to provide both expected values and uncertainty estimates for predictions, as well as its proven performance in a previous AL study on the TYK2 target by Thompson et al.^21^ GP is a nonparametric Bayesian approach that provides a probabilistic framework for making predictions. GPs make use of a kernel function to measure the similarity between the data points. The kernel function is used to construct a covariance matrix of observed features, which is used to make predictions for the unseen data. In our implementation, we use the Tanimoto similarity kernel, which is particularly useful for measuring the similarity between sets, making it suitable for binary fingerprints. Our implementation of GP is based on the GPyTorch version 1.10^31^ library.

By default, ligands were featurized using ECFP8 fingerprints from OpenEye’s OEChem toolkit version 3.4.0.0.^32^ To assess the influence of chiral descriptors on the GP model performance, an alternative featurization using Morgan fingerprints with chirality descriptors was also assessed as a part of benchmarking. Morgan fingerprints were generated using RDkit^33^ and have a radius of 4. Both ECFP8 and Morgan fingerprints were hashed to 4096 bits.

#### Chemprop

CP is a message-passing neural network designed for molecular property prediction. The model operates by iteratively updating the atom and bond features of a molecule through message-passing layers (and hence does not rely on molecular fingerprints, such as GP). This allows the model to capture both local and global structural information. CP has been shown to perform well on a variety of molecular property prediction tasks, including solubility, toxicity, and binding affinity.^34^ Monte Carlo dropout^35^ was used to provide a measure of uncertainty. Our implementation is based on the Chemprop package version 1.6.1,^36^ and we use a model pretrained on potency data across 1788 targets, including Kinases, GPCRs, and Proteases. The data for pretraining the model are mostly taken from ChEMBL^26^ for targets having more than 200 interaction data points. For our work, we unfreeze the entire encoder and train the model in each AL cycle for 500 epochs with a batch size of 50 and a learning rate of 0.0001 for 10 warmup epochs, ramping up to a maximum of 0.001 for the remaining epochs using a Noam scheduler. These hyperparameters for fine-tuning the CP model are empirically determined.

### Analysis

We used a range of metrics to evaluate the performance of our AL benchmark. These metrics are selected to assess both the exploitative and predictive aspects of the model. Whereas exploitation is governed by the predictive prowess of the model on the high end of the potency range, regression metrics reflect its performance on the bulk of the data.

To define Recall and F1 scores in this context, the selection process is converted to a classification task. A compound is considered “True” if it has been acquired for labeling in any of the previous AL cycles and “False” otherwise. We further categorize compounds into “active” and “inactive” based on their relative ordering, specifically focusing on the top 2% and top 5% of compounds. The Recall metric is calculated according to eq 2
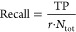
2Here, TP represents the number of true positives, *r* is the fraction of compounds considered true (either 0.02 for the top 2% or 0.05 for the top 5%), and *N*_tot_ is the total number of samples in the given data set. The expectation value of the TP for a random selection is given by *r N*_acq_, where *N*_acq_ represents the number of compounds acquired or selected in the current AL cycle. The F1 score is particularly useful for assessing the model’s ability to correctly identify the most promising compounds (TP) while minimizing the selection of false positives (FP). We compute the F1 score according to eq 3
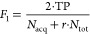
3Here, we used the relations *N*_acq_ = TP + FP and *r N*_tot_ = TP + FN. False negatives (FN) represent the promising compounds that the model failed to identify.

To assess the predictive power of the models, we use coefficient of determination R2, Spearman ρ, and the root-mean-square error (RMSE). To visualize the high-dimensional fingerprints on two-dimensional maps, we use UMAP^24^ as a dimensionality reduction technique using the umap-learn package version 0.5.3.^37^ In contrast to widely used tSNE plots, UMAPs do not rely on a fixed cutoff and instead keep the number of neighbors constant (here, 50), which is an arguably better choice for preserving the global structure of the data.

## Results and Discussion

### Model Benchmarking

**Figure 3:**
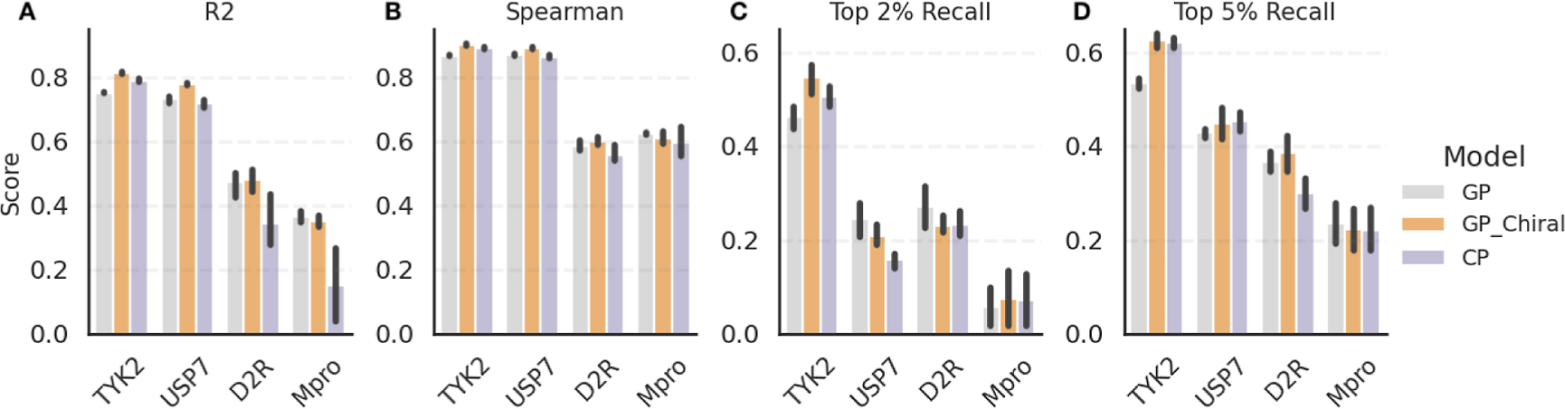
Benchmarking GP and CP models on four target data sets (TYK2, USP7, D2R, Mpro) used in our AL study. We use 20% of the data set for training the models and the remaining 80% as a test set to calculate the metrics. (A) R2, (B) Spearman ρ, (C) Recall for top 2% compounds, and (D) Recall for top 5% compounds. Data sets are sorted based on their size, i.e., from large to small (see also Table 1). While both models exhibit robust predictive power, the CP model is more sensitive to the data set size than the GP model, and the introduction of chirality descriptors offers limited enhancement in model accuracy.

Figure 3 compares GP and CP models trained on a 5-fold split, where the training set was made up of 20% of the data and metrics were calculated on the test sets containing the remaining 80%. Error bars represent a 95% confidence interval across these folds. The calculated metrics are the coefficient of determination R2 (A), Spearman ρ (B), and the Recall of top 2% (C) and 5% (D) samples across the test sets. To evaluate the impact of chiral molecules, we considered a GP model trained on Morgan fingerprints with chirality descriptors in addition to achiral ECFP8 fingerprints with the same size and radius.

The goal of this work is to assess the performance of the two models on each data set in the limit of a large training set. However, the training sets amount to a fraction of the pool rather than being comparable in size to reflect the total number of compounds typically acquired in AL. Table 1 shows that the subsequent AL experiments acquire less than 20% of the data in total except for Mpro, where over half of the data are acquired. Given the lack of bias in a random sample selection, this analysis provides an upper bound for the model performance in terms of regression. It also gives an estimate for the Recall given a large amount of training data.

For each data set, all of the models demonstrated predictive capabilities, with an R2 larger than 0.3 (with the exception of CP trained on Mpro data), Spearman ρ over 0.5, and top 5% Recalls of 0.2 or more. This suggests that it is possible to train a predictive model even on the more challenging data sets given enough training data sampled broadly over the available chemical space. There is, however, a clear trend in model performance concerning the data set size. R2 and top 5% Recall monotonously decrease with decreasing size of the data set. The trend is also present, to a weaker extent, for Spearman ρ and the top 2% Recall, which is more pronounced for CP compared to that for GP. This observation aligns with the understanding that CP, being a deep learning model, benefits from larger data sets.

Both Mpro and D2R data sets presented challenges due to their heterogeneous nature. The lack of distinct clustering in the UMAP projections (Figure 1C,E) for these data sets, especially for D2R, indicates the diverse composition of compounds, making them potentially harder to fit with ML models. Spearman ρ for these two data sets is indeed lower than those for TYK2 and USP7. However, the comparably large training set accounts for diversity to some degree, which is why differences between different types of data sets are not very pronounced in this benchmark.

The GP model using chirality descriptors showed comparable performance to the model trained on achiral fingerprints, suggesting that introducing chirality representation did not significantly enhance the models’ performance. For Mpro and D2R compounds, which have a significant number of chiral centers, the performance remained comparable between both the fingerprints. Similarly, the USP7 data set, which contains only a few chiral compounds, showed no significant improvement with the chirality descriptors. It is likely that chirality does not play an important role in the structure–activity relationship of these series or that stereochemistry information is missing for some of the data (such as TYK2).

### AL Strategies

In this section, we investigate the impact of batch sizes, sample acquisition strategies, and the exploration–exploitation trade-off in simulated AL scenarios. The focus herein lies on protocols with a distinct exploration phase, which aims to select diverse samples for building a robust and predictive model, followed by greedy acquisition using the merit predicted by the model (exploitation). Splitting the AL protocol into these two stages simplifies the evaluation of the individual benefits of each phase. Note, however, that the interleaving of the two strategies has also been studied in literature studies^5,20,23^ yielding good results. We always acquire a total of 360 compounds for labeling to fairly evaluate selection protocols that differ in their batch sizes.

#### Selection of Initial Samples

In an AL setting, it is essential to understand the influence of the initial sample selection strategy as it guides the trajectory of subsequent AL cycles. We explore initial batch sizes and strategies for the selection of these compounds using three distinct selection protocols. All of them are initiated with 60 samples selected at random (following the suggestion from Thompson et al.^21^ in the TYK2 study) and use a batch size of 30 compounds for exploitation. The “random–exploit” protocol immediately switches to exploitation from the first cycle on. Both the “random–explore–exploit” and “random–random–exploit” protocols acquire 60 more compounds after the initial selection with the purpose of improving coverage of the chemical space, resulting in an effective initial batch size of 120. More precisely, the “random–random–exploit” protocol selects the additional 60 compounds at random, whereas the “random–explore–exploit” protocol utilizes the model’s prediction uncertainty to select the additional compounds over two exploration cycles. Due to the constraint on the total number of acquired compounds, the “random–explore–exploit” and “random–random-exploit” protocols acquire fewer compounds over the exploitation phase than “random–exploit”.

**Figure 4:**
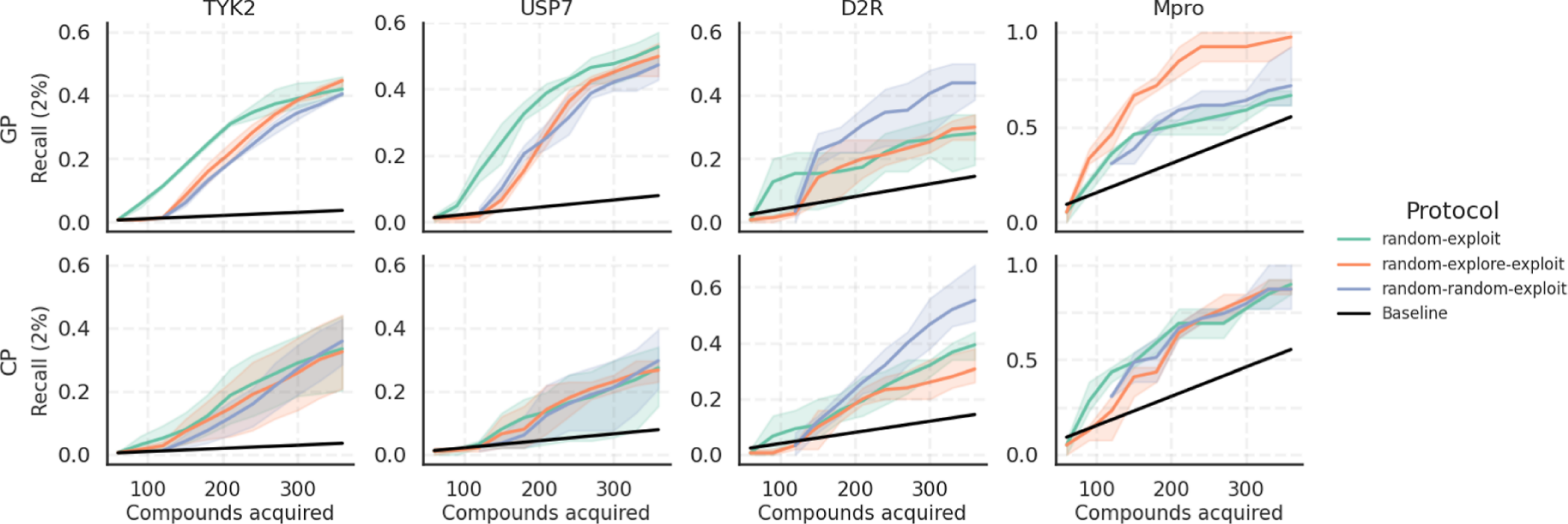
Top 2% Recall achieved with different AL protocols for initial sample selection across the four target data sets. The “random–exploit” protocol acquires 60 compounds at random before switching to exploitation, “random–explore–exploit” acquires another 60 compounds using the prediction uncertainty following the initial batch selected at random, and “random–random–exploit” starts with 120 compounds selected at random. The shaded area displays the variance over three repeats initialized with a different random seed for the initial sample selection. The baseline shows the expectation value for the Recall upon random acquisition. The protocol yielding the best Recall is consistent between the two models for each data set but not between different data sets.

**Figure 5:**
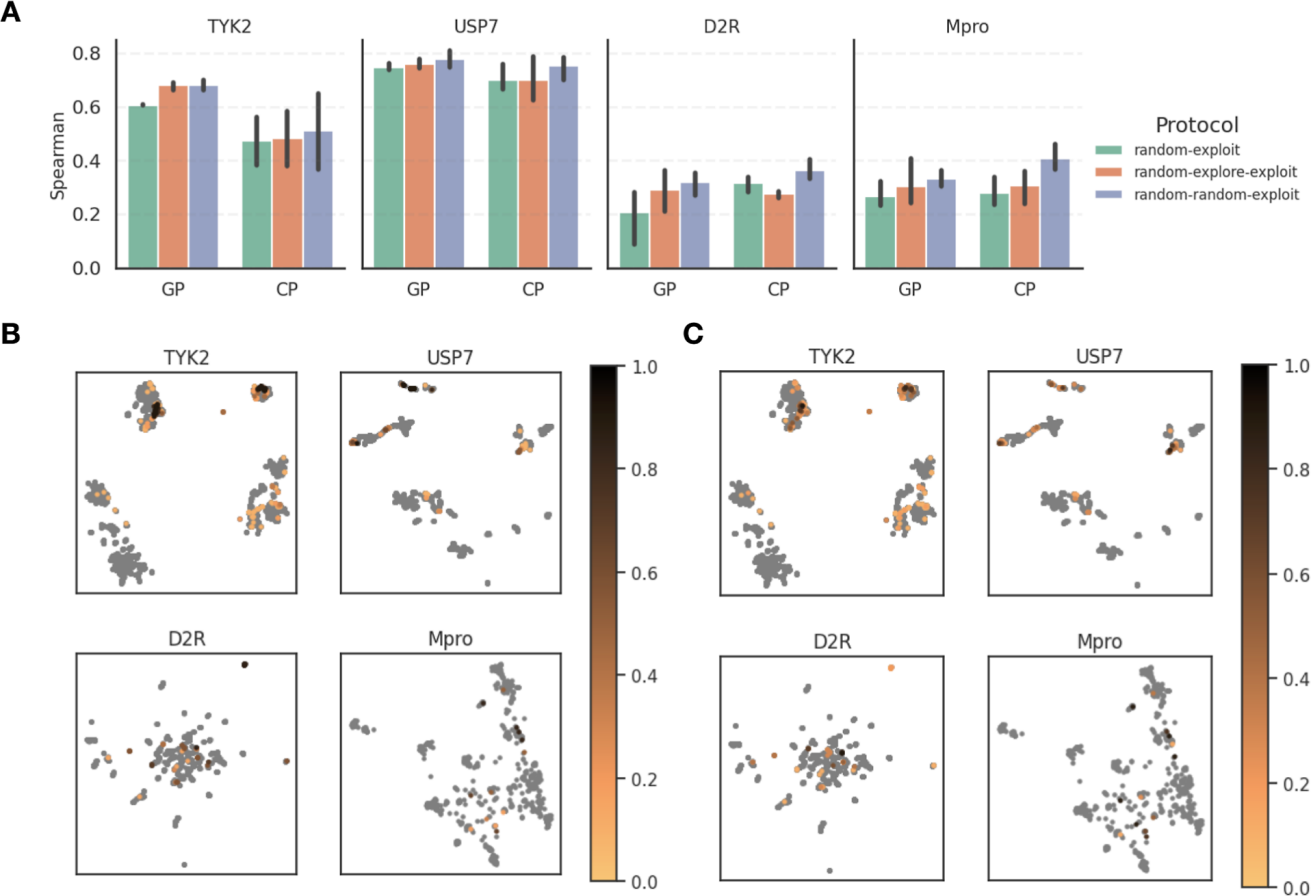
Evaluation of model performance with Spearman ρ and UMAPs showing compound acquisition across AL protocols. (A) Spearman ρ of the final models trained on all 360 selected compounds, emphasizing the enhanced predictability with larger initial batch sizes of 120 in the “random–explore–exploit” and “random–random–exploit” protocols. (B) UMAP projection of the GP model selection displaying the top 2% compounds in each data set, colored by the frequency of their acquisition. UMAP shows consistent acquisition of compounds in dense clusters and the challenge of identifying sparse clusters. (C) UMAP of the CP model, highlighting similar trends in compound acquisition. Overall, we can see that the GP model is more consistent in compound selection than the CP model across multiple AL runs.

Figure 4 shows the top 2% Recall (eq 2) across the four data sets for the three protocols and both GP and CP models, and the top 5% Recall is shown in Figure S2. Figure 5A shows the corresponding Spearman ρ values of the final models trained on all 360 selected compounds. A more detailed plot showing the Spearman ρ as a function of the number of compounds acquired can be found in Figure S3, and the equivalent plots for the R2 and the RMSE are also shown in Figures S4 and S5, respectively. A general observation is that a larger initial batch size of 120 with the “random–explore–exploit” and “random–random–exploit” protocols yields more predictive models and therefore augments the likelihood of pinpointing active compounds, as evidenced by the higher Spearman ρ (Figure 5A) and larger slopes in the Recall curves (Figures 4 and S2). The same trend is also seen for R2 and RMSE in Figures S4 and S5, respectively. However, the increase in the initial batch size comes at the expense of an increased initial training cost for the model. The F1 score (Figures S6 and S7) decreases in later cycles for all data sets except TYK2 in response to diminishing precision, which is indicative of an increased cost per identified top compound. This decline is more pronounced for smaller data sets.

For the TYK2 and USP7 data sets, a smaller initial batch size was found to be more adequate with GP, given that the explorative protocols only caught up after around 300 acquired compounds. In contrast, the more heterogeneous D2R and Mpro data sets greatly benefited from a larger initial batch. As can be seen from Figure S1, D2R predominantly contains top compounds in small dense clusters, while Mpro’s top compounds are more dispersed as singletons. This distinction can explain the opposite trends in terms of selection protocols for these two data sets. However, such nuances are not known a priori and cannot be used to inform the choice of the exploration method. The drop of predictive power (Figures S2 and S4) on Mpro over the course of AL is associated with the small size of this data set compared to the number of acquired training samples as well as the exploitative acquisition, which causes a potency imbalance between training and pool data. In comparison to the GP model, the CP model underperformed on all data sets except D2R, although its predictive performance tends to be more consistent between the three protocols. By contrast, CP outperformed GP on D2R, where the underlying global model is likely to account well for the diversity of these compounds.

Figure 5B,C shows UMAPs where the top 2% compounds in each data set are colored by the frequency of their acquisition (averaged over different protocols and random seeds for initialization) using GP or CP, respectively. A breakdown by protocol for each data set can also be found in Figures S8–S11, which shows that there is little difference between compounds identified by different protocols. A common trend in Figure 5B,C is a consistent acquisition of compounds in dense clusters, while sparse clusters remain difficult to track with both models. Overall, we could see that the selection with the GP model is slightly more consistent across multiple AL runs.

We also evaluated the performance of AL relative to training models on a larger proportion of the data, as was the case in the Model Benchmarking section. The recall achieved by AL using a smaller fraction of the data (Figure 4) is comparable to or better than the models trained in a single batch on 20% of each data set (Figure 3), demonstrating the benefits of partitioning the training process into multiple stages. On the other hand, R2 and Spearman ρ are lower for models constructed using AL (except for the Mpro data set due to its size). It might be tempting to conclude that the size of the training set is the deciding factor for a model’s predictive quality, but this is not entirely true. Figures S3 and S4 show that the R2 and Spearman ρ stop improving after the start of the exploitation phase, and the effective performance of the final models is comparable to that of the models constructed only from the initial 60 or 120 samples. Similarly, the models constructed from a large randomly chosen batch better account for the variety in the data than samples selected using an exploitative strategy. As stated before, the amount of diversity in the data is strongly linked to the speed of convergence of a model with an increasing number of training samples. On the TYK2 and USP7 data sets, GP models trained on 360 samples reach more than 70% of the rank-ordering performance than that of corresponding models trained on more than twice as many samples (CP underperforms on TYK2, reaching only about 50% of the performance of the larger model). By contrast, this ratio drops to 30–50% for D2R, which also benefited greatly from an increase in the initial batch size. In summary, AL yields greater benefits for compound pools, which display a high degree of similarity and strong structure–activity relationships.

#### Batch Size for Exploitation

To systematically investigate the influence of the batch size in exploitation cycles, we fixed the initial sample selection at 60 samples chosen randomly and the subsequent 60 samples selected based on the exploration strategy. We then evaluated four distinct batch sizes for the subsequent AL cycles, namely, 20, 30, 60, and 120. Given our constraint of acquiring a total of 360 compounds, smaller batch sizes necessitate a greater number of AL cycles to reach this total. The protocols varied in batch sizes, with “batch-size-20” using three exploration batches of 20 each followed by 12 exploitation batches of 20, “batch-size-30” using two exploration and eight exploitation batches of 30, “batch-size-60” using one exploration and four exploitation batches of 60, and “batch-size-120” combining one exploration batch of 60 with two exploitation batches of 120.

**Figure 6:**
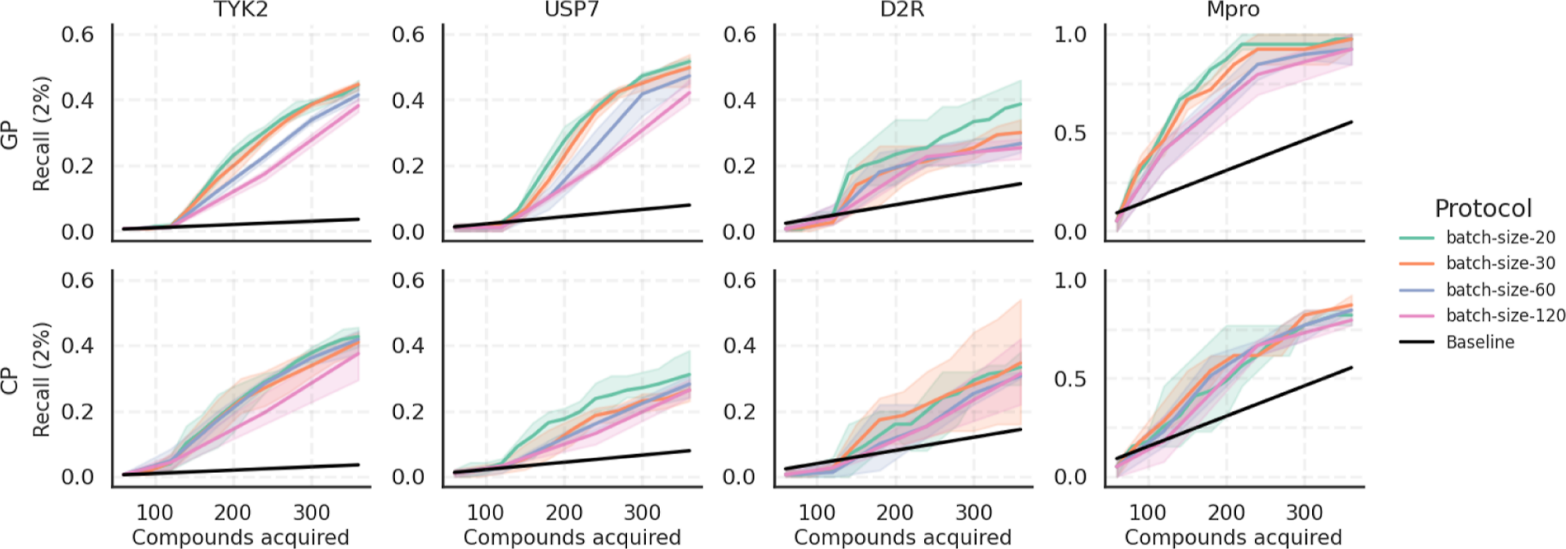
Comparison of the top 2% Recall across AL protocols varying in their batch size. The “batch-size-20” protocol uses three exploration batches and 12 exploitation batches of 20; “batch-size-30” employs two exploration and eight exploitation batches of 30; “batch-size-60” employs a single exploration and four exploitation batches of 60; and “batch-size-120” employs one exploration batch of 60 and two exploitation batches of 120. The comparison suggests that the Recall improves with decreasing batch size across all data sets with both models.

Figure 6 shows the top 2% Recall across different batch sizes. We can see a clear trend where smaller batch sizes consistently outperform larger ones across all data sets, and this holds true for both GP and CP models. The same trend can be seen in Figure S12, which shows the top 5% Recall, and also considering F1 scores (Figures S13 and S14), indicating that smaller batch sizes favor both precision and recall. The same trends are reflected by the metrics Spearman ρ, R2, and RMSE across all data sets, except for Mpro (as seen in Figures S15–S17). This trend can be understood by considering the ratio of pool and training sizes. When the pool of available data significantly outnumbers the training set, the model benefits from small, incremental additions to its training data, making a batch size of 1 theoretically optimal. This is because each new data point refines the models understanding. However, in practical scenarios, using extremely small batch sizes might not be feasible as it would require acquiring potency data in a time-consuming serial manner rather than a more efficient parallel approach. This advantage of smaller batches diminishes when the pool size is roughly equal to the training size, where the predictive power of a model decreases upon imbalancing the data by using an exploitative acquisition strategy.

### Modeling Noise on Labels

**Figure 7:**
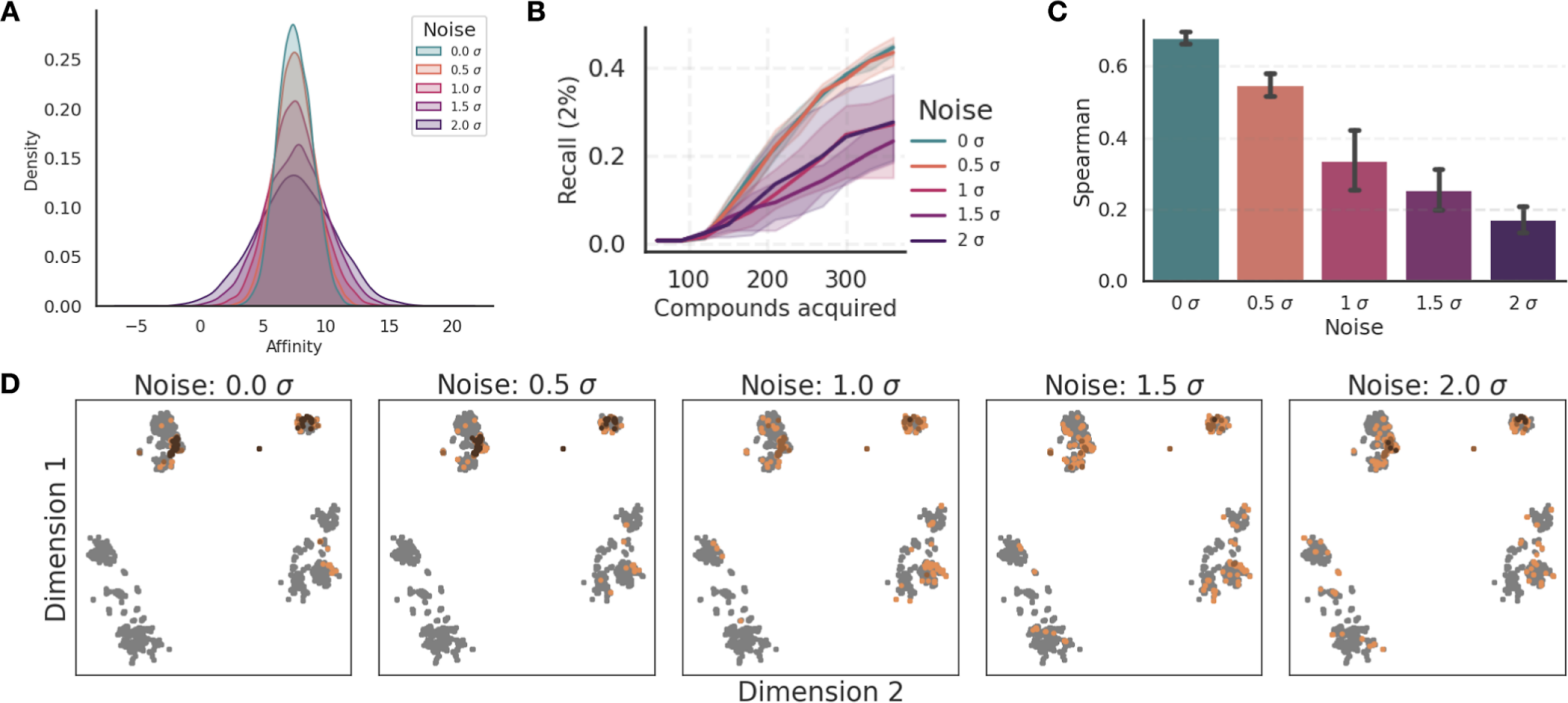
Analysis of the influence of Gaussian noise on the outcomes of AL using the GP model on the TYK2 data set. The standard deviation of the added Gaussian noise was scaled with respect to the standard deviation of TYK2 p*K*_i_ values, with factors ranging from 0 (no noise) to 2. (A) Kernel density estimation plot of the p*K*_i_ distribution across varying noise magnitudes. (B) Top 2% Recall, highlighting a noticeable decline at increased noise levels. (C) Spearman ρ revealing diminished model predictability with increasing noise. (D) UMAP visualization of the compounds selected in the exploitation phase, colored by the acquisition frequency across three distinct AL iterations with randomized initializations. The UMAPs emphasize the AL framework’s capability to consistently identify top-binding compound clusters, even amidst noise interference.

The measurement of binding affinities by experimental and computational means is subject to noise although being fed to the model as “true” values. To systematically study the influence of noise in training data, we introduced Gaussian noise to each data set. The noise was generated by sampling random numbers from a Gaussian distribution with a mean of zero and a standard deviation (σ) ranging from zero to two times the standard deviation of the underlying potency data, meaning that the amounts of noise vary between data sets in absolute terms. Our investigation considers the noise multipliers 0, 0.5, 1, 1.5, and 2. Similar to the batch size modulation, we fixed the initial sample selection at 60 samples chosen randomly and the subsequent 60 samples selected by using the exploration strategy. We ran the AL pipeline three times with different random seeds for selection of the initial batch. The transformation of the potency distribution at each noise level for the TYK2 data set is visually presented in Figure 7A. Similar transformations for other data sets, namely, USP7, D2R, and Mpro, are shown in Figures S18A, S19A, and S20A, respectively, and the UMAPs of the noisy data are shown in Figure S21. An overarching observation from Figure S21 is that the introduction of stochastic noise retains the macroscopic structure of the clusters while smoothing out the microscopic features within these clusters across all target data sets.

The introduction of noise has a pronounced effect on the regression performance and Recall of the constructed model. As depicted in Figure 7B, the top 2% Recall rapidly decays when the noise level exceeds 1 σ. This trend of declining Recall with increasing noise is consistently observed across other data sets, as shown in Figure S18B for USP7, Figure S19B for D2R, and Figure S20B for Mpro.

Furthermore, Spearman ρ drops with increasing noise levels, as shown in Figure 7C. However, the models remain predictive even with high amounts of noise, maintaining a positive Spearman ρ coefficient. This trend also holds true for USP7 and D2R (Figures S18C and S19C) but not for Mpro, where Spearman ρ drops below zero for a noise addition of 2 σ, as shown in Figure S20C.

AL is relatively consistent in identifying the top binders between independent repeats even under noisy conditions. Figure 7D shows compounds acquired in the exploitation phase colored by the fraction of three AL repeats that selected them. Similar observations can be made for other data sets, as shown in Figure S18D for USP7, Figure S19D for D2R, and Figure S20D for Mpro. These findings are consistent with the work of Bellamy et al.^38^ for synthetic affinity data, who found a better Recall in terms of the true (noiseless) labels than considering the noisy values used to train the model. This robustness underscores the fact that while noise may introduce perturbations, learning the overarching structure–activity relationship trends remains preserved. Interestingly, the presence of noise might even be beneficial for exploration, aiding in overcoming potency barriers in the chemical space. However, while noise can enhance exploration, it hampers the exploitative power of the model.

A similar trend of decaying Recall with increasing noise also is observed for the CP model, as depicted in Figure S22. Spearman ρ for the CP model also exhibits a decline with increasing noise levels (Figure S23). However, the CP model is far less consistent in identifying relevant clusters compared to the GP model in the presence of noise (Figure S24), especially for TYK2 and USP7.

In conclusion, while the presence of stochastic noise impairs the performance of AL, the models demonstrate a commendable ability to identify large-scale trends. However, the Gaussian noise simulation in this study primarily accounts for stochastic noise. Systematic errors are not accounted for. They can particularly be detrimental, as they introduce a bias in the activity landscape, potentially misguiding the AL process. One approach to investigating the effect of systematic bias in AL could involve SMARTS filtering to isolate compounds with specific functional groups and then introduce noise with a nonzero mean.

## Conclusions and Outlook

We comprehensively evaluated the effect of different parameters for AL protocols based on a range of metrics. These capture the identification of top binders, the predictive quality of the underlying ML model, and a qualitative analysis of the identified clusters in the chemical space. Simulating identical AL runs on different binding affinity data sets allowed us to assess different aspects of the AL strategies, as well as to capture trends with respect to the size and composition of a data set.

Using RBFE to label a chemical library of 5000 compounds, each calculation averaging 8 GPU hours at a rate of $2.50 per GPU hour, results in a total cost of $100 K. Using AL and RBFE to label just 300 compounds incurs a cost of $6k and can yield a comparably predictive model. AL can identify top binders using a significantly smaller fraction of the data to train. We observed a pronounced dependence on the data set size, although the diversity of compounds in a data set is the most decisive factor contributing to the margin of profit achievable by AL. This may be reduced even further with reliable docking or ML labeling protocols in the future. A similar trend was identified when varying the initial batch size for AL, where the more diverse D2R and Mpro data sets benefited from a large initial exploration phase compared to TYK2 and USP7.

The results suggest that certain design strategies will help in designing the most useful AL protocols. These findings have implications for the design of ligand pools for AL. The TYK2 and USP7 data sets, which consistently led to predictive models and a high Recall, are made up of few distinct scaffolds and a large number of substitutions or even a combinatorial enumeration of substituents in the case of TYK2. The confinement of chemical space is essential for a small number of samples to represent a sizable pool. In practice, however, a combinatorial exploration of substituents is not always desirable when a strict filtering of compounds is necessary due to constraints on the physicochemical properties. In such cases, increasing the batch size is a more defensive approach to achieve success with AL. In the present study, using the model uncertainty in the initial exploration phase did not yield any observable benefits over selecting the compounds at random.

For the exploitation phase, our findings consistently suggest that training in small batches results in the highest Recall. The performance gains, however, are incremental when the batch size is reduced below 30 samples. Very small batches are also undesirable from a practical perspective due to the increase in the number of AL cycles and the overall turnaround time.

The addition of noise to potency data was found to have detrimental effects on the exploitative power of AL if the variance of the noise is equal to or larger than the variance of the affinity values. On the other hand, the noise did not prevent the GP model from finding large-scale active regions in the chemical space, even when its variance exceeded the underlying signal. The CP model was more affected by noise in the data and lost its predictive power with high levels of noise. Together with the fact that CP outperformed GP on the AL runs for D2R, whereas GP performed better in the general case, it is likely to be more sensitive to the local structure of chemical space and, at the same time, is more vulnerable to noise.

Using a Gaussian model noise is a drastic simplification to account for the entirety of errors that may be introduced by the labeling methods, such as RBFE, and understanding their nature for a given ligand series is crucial. A validation prior to running AL with the chosen labeling method (e.g., AL–RBFE) is highly recommended, as it may not only provide insight into the magnitude of stochastic errors relative to the dynamic range of RBFE values but also reveal systematic offsets for certain functional groups, which can alter the entire course of an AL run. One aspect to look at in the future is data initialization beyond starting with 60 random samples consistently. Depending on the data set, preclustering data and selecting randomly from clusters may be desirable. This will introduce additional choices around data representation and clustering method choice [e.g., using density-based spatial clustering of applications with noise (DBSCAN) on UMAP data]. However, depending on the data set, clustering may not be helpful. For example, varying ε for DBSCAN in Figure S31 does not give good clusters for D2R but may give rise to better results for, e.g., Tyk2. As only some data sets show clear clusters, using just random selection is the most straightforward approach when investigating different data sets and will avoid any bias introduced from clustering. Overall, understanding the relationships between data, models, and selection strategies in AL pipelines paves the way for establishing protocols for choosing these parameters in an automated fashion.

## Data Availability

All data for the experiments carried out can be found at https://github.com/meyresearch/ActiveLearning_BindingAffinity.

## Supporting Information Available

Data set analysis and additional experimental results (PDF)
